# Supplementary material for: Causal interplay between lactose intolerance and gut microbiota: a combined bidirectional Mendelian randomization and in vivo validation study
Source: Front Nutr. 2026 Jun 1;13:1803337. doi: 10.3389/fnut.2026.1803337 (PMC13265576; doi:10.3389/fnut.2026.1803337)
Supplement: Supplementary file 1 [file Data_Sheet_1.zip › supplementary materials/Forward/leave one out cross validation/ebi-a-GCST90027475.finngen_R12_E4_LACTONAS.pdf]

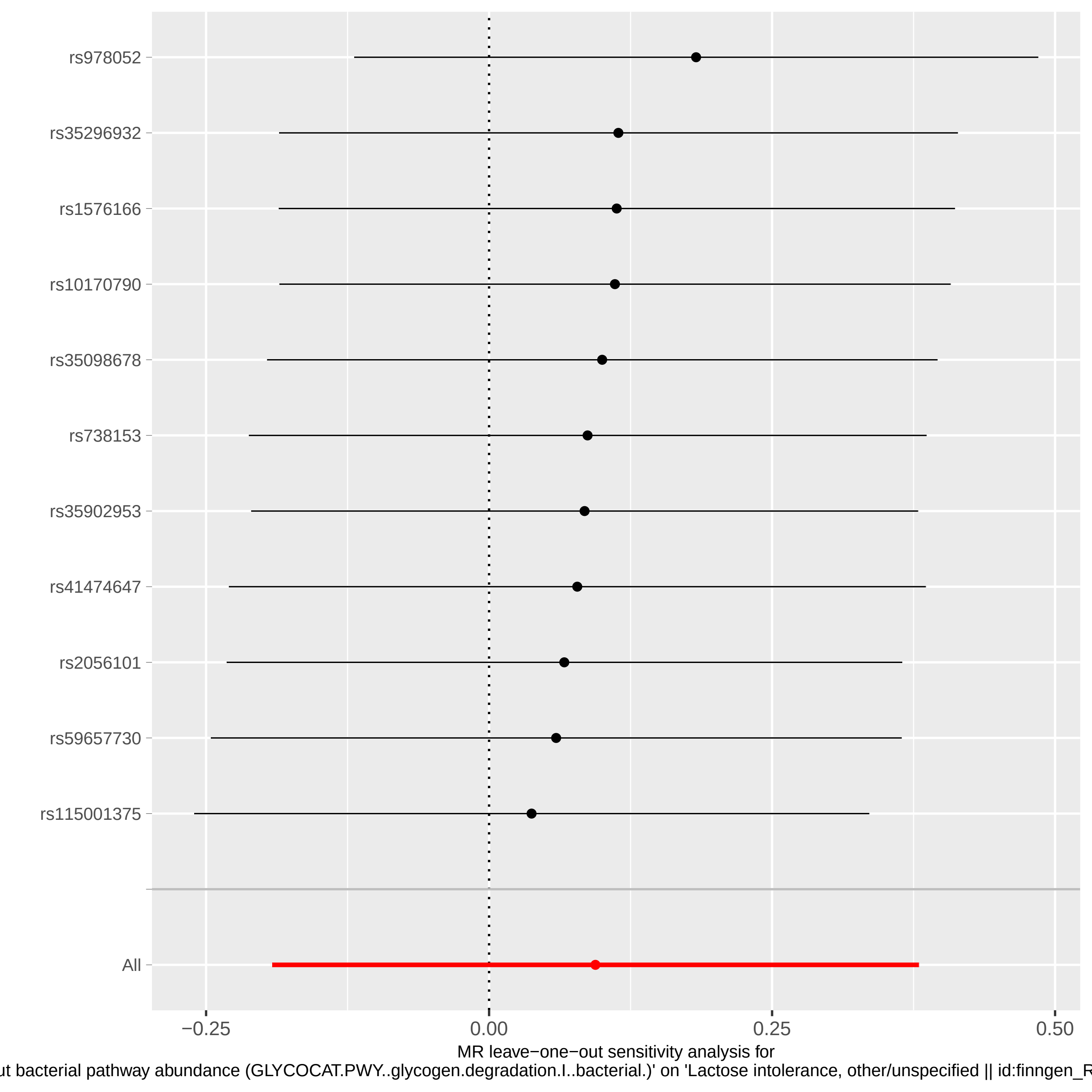
rs978052
rs35296932
rs1576166
rs10170790
rs35098678
rs738153
rs35902953
rs41474647
rs2056101
rs59657730
rs115001375
All
−0.25
0.00
0.25
0.50
MR leave−one−out sensitivity analysis for
ut bacterial pathway abundance (GLYCOCAT.PWY..glycogen.degradation.I..bacterial.)' on 'Lactose intolerance, other/unspecified || id:finngen_R
